# Atypical Manifestation of DRESS Syndrome

**DOI:** 10.1155/2020/6863582

**Published:** 2020-04-12

**Authors:** Christopher Hakim, Constantine Melitas, Eric Nguyen, Kha Ngo

**Affiliations:** ^1^Department of Internal Medicine, Ascension Providence Hospital, Michigan State University College of Human Medicine, Southfield, USA; ^2^Department of Gastroenterology, Ascension Providence Hospital, Michigan State University College of Human Medicine, Southfield, USA; ^3^American University of the Caribbean School of Medicine, Coral Gables, USA

## Abstract

The differential for liver transaminases over 1000 units/liter typically includes liver ischemia, acute viral hepatitis, acetaminophen toxicity, and autoimmune hepatitis. Prompt evaluation is imperative as these etiologies can lead to fulminant liver failure. We present a case of transaminases over 1000 units/liter from an atypical etiology. A 52-year-old male, previously treated with allopurinol for an acute gout flare, presented with persistent fevers. Given that he had taken a “high-risk medication” 2–6 weeks before presentation, subsequently presented with fever, rash, renal impairment, elevated liver enzymes in the thousands, and peripheral eosinophilia, DRESS syndrome secondary to allopurinol was diagnosed.

## 1. Introduction

Elevated liver chemistries (LFTs) include a wide differential diagnosis, and delineating the cause is imperative for treatment. More specifically, LFTs in the thousands include a narrow differential in which assessment for acetaminophen toxicity and testing for acute viral causes, autoimmune hepatitis, ischemic hepatitis/shock liver, and vascular disorders including acute portal vein thrombosis and acute Wilsonian crisis, as well as workup for biliary obstruction, must be performed [1].

We present a case of allopurinol-induced drug reaction with eosinophilia and systemic symptoms (DRESS) syndrome, which presented as a rare cause of liver enzymes in the thousands.

## 2. Case Report

A 52-year-old African American male presented to the emergency department from his primary care physician's office due to persistent fever. The patient had been discharged from the hospital one week prior after being hospitalized for diarrhea due to *Clostridium difficile* colitis, which was treated with oral vancomycin. During that hospitalization, he was started on allopurinol for an acute gout flare after which he developed a generalized purpuric, morbilliform rash, which started on his chest and spread to his extremities. He was discharged on a prednisone taper for a suspected drug rash, which he completed prior to returning to the hospital.

His past medical history includes hypertension, chronic kidney disease stage 5, osteoarthritis, GERD, and gout. On initial examination, his temperature was 38.3°C with vitals within normal limits. Skin excoriations were noted, but no rash or lymphadenopathy. His cardiopulmonary and abdominal exams were unremarkable. Laboratory data demonstrated a white blood cell count within normal limits, hemoglobin 7.9 gm/dL, platelet count 71K/mcl, creatinine 6.4 mg/dL (baseline 5-6 mg/dL), BUN 60 mg/dL, and elevated LFTs with AST 878 units/L, ALT 1,117 units/L, alkaline phosphatase 169 units/L, and GGT 209 units/L. Of note, the patient had known anemia of chronic disease; however, his thrombocytopenia was a new occurrence. Lactic acid was normal, and urinalysis showed no signs of active urinary tract infection. Chest X-ray demonstrated no acute processes.

During hospitalization, his LFTs continued to increase and thrombocytopenia worsened. The gastroenterology service was consulted on the second day of hospitalization. His temperature of 38.7°C persisted. His LFTs remained elevated with AST 676 units/L, ALT 1,262 units/L, alkaline phosphatase 173 units/L, and total bilirubin 0.6 mg/dl with a normal prothrombin time.

Despite a negative acetaminophen level, *N*-acetylcysteine (NAC) was administered via three consecutive doses given over 21 hours with subsequent decrease in LFTs. Viral serologies (hepatitis A/B/C, EBV, CMV, HSV, and VZV), autoimmune studies (IgG, ANA, smooth muscle antibody, and mitochondrial antibody), and ceruloplasmin were normal as well as serum electrolytes, thyroid function tests, ammonia level, and stool studies. Abdominal ultrasound with doppler demonstrated hepatomegaly without evidence of vascular occlusion or biliary abnormalities.

On the third day, he developed peripheral eosinophilia (13%). Given that he had taken allopurinol two weeks before presentation, subsequently presented with fevers, rash, elevated LFTs, and peripheral eosinophilia, with an extensive workup negative for alternative processes, DRESS syndrome was suspected. LFTs continued to improve significantly. The remainder of testing was negative including infectious workup, alternative autoimmune and genetic etiologies, and toxicology screening leading to discharge on day seven.

## 3. Discussion

DRESS syndrome is a hypersensitivity drug reaction characterized by rash, hematologic abnormalities, lymphadenopathy, and organ involvement, including liver and kidney with mortality as high as 10% [2, 3]. It has a prevalence of 2.18 per 100,000 patients with specific diagnostic criteria [4, 5]. The pathogenesis is hypothesized to be secondary to an accumulation of toxic drug metabolites due to various enzymatic deficiencies [2].

The presenting symptoms in almost all patients include skin rash, liver involvement, hypereosinophilia, and lymphadenopathy [6]. The first symptoms to present are fever and rash, as were seen in our patient prior to his admission. Skin manifestations occur as a morbilliform eruption, which involves over 50% the body and includes two or more of the following: facial edema, infiltrative lesions, scaling, and/or purpura [6].

The liver is the most common affected organ in DRESS syndrome. Liver involvement is generally mild and infrequently causes liver chemistry elevations in the thousands with rare progression to fulminant hepatic failure [7]. Hematological findings are also commonly seen in DRESS syndrome, with eosinophilia being the most common manifestation, often delayed by 1-2 weeks [6, 8, 9]. Renal dysfunction is also commonly appreciated [8].

Allopurinol has one of the strongest associations with DRESS syndrome [10]. Other high-risk medications include carbamazepine, lamotrigine, phenytoin, and phenobarbital [5].

Diagnosing DRESS syndrome can be challenging but should be suspected in a patient who received a new high-risk medication in the previous 2–6 weeks and presents with characteristic symptoms as outlined above. Bocquet's diagnostic criteria include the presence of three conditions such as drug-induced skin eruption, eosinophilia ≥1500/mm, and at least one of the following systemic abnormalities: lymphadenopathy, hepatitis (LFTs >2 ULN), interstitial nephropathy, interstitial lung disease, or myocardial involvement [5]. The RegisSCAR criteria for diagnosis is another diagnostic tool which classified our patient as “probable” of having DRESS syndrome [11]. Additionally, there is a Japanese diagnostic tool which has been shown to be significantly less accurate [12].

Supportive therapy and prompt withdrawal of the offending agent is the primary treatment. Most patients recover completely in weeks to months after drug withdrawal. Systemic corticosteroids are used by some; however, the literature has not shown a significant benefit [13].

In the setting of significant liver injury, administration of NAC and transfer to a liver transplant center are important considerations. NAC improves overall tissue oxygen delivery to the liver and has been shown to improve mortality. Additionally, studies have demonstrated an increase in transplant-free survival and post-liver transplant survival in several causes of nonacetaminophen acute liver injuries including drug-induced, viral, and autoimmune [14–16].

To avoid complications and death due to DRESS syndrome, prompt diagnosis with removal of the offending agent, supportive care, and administration of NAC in the setting of significant liver injury are imperative.
